# Genome-Wide Identification and Expression Analysis of *PaNRT* Gene Family Under Various Nitrogen Conditions in Avocado (*Persea americana* Mill.)

**DOI:** 10.3390/genes15121600

**Published:** 2024-12-14

**Authors:** Yuan Tian, Ruiyuan Jiang, Jian Qin

**Affiliations:** 1 College of Landscape Architecture, Nanjing Forestry University, Nanjing 210037, China; tianyuan@njfu.edu.cn; 2 Institute of Fruit Tree Research, Guangdong Academy of Agricultural Sciences, Key Laboratory of South Subtropical Fruit Biology and Genetic Resource Utilization, Ministry of Agriculture and Rural Affairs, Guangdong Provincial Key Laboratory of Science and Technology Research on Fruit Trees, Guangzhou 510640, China

**Keywords:** avocado, *PaNRTs*, nitrogen, expression levels

## Abstract

**Background:** Avocado is an important economic fruit tree that requires a lot of nitrogen (N) to support growth and development. Nitrate transporter (NRT) gene family plays an essential role in N uptake and use in plants. However, no systematic identification of the NRT gene family has been reported in avocado. **Methods:** Bioinformatic analysis was used to identify and characterize the NRT gene family in avocado. The five N additions (29.75, 59.50, 119.00, 178.50, and 238.00 mg/L N) were used to identify the N requirement of avocado seedlings based on physiological indexes, while RNA-seq was conducted to analyze the response of *PaNRTs* under low-N and high-N conditions. **Results:** Sixty-one members of the NRT gene family were identified and dispersed on 12 chromosomes in avocado. Many cis-regulatory elements (CREs) related to phytohormonal and stress response were found in the *PaNRTs* promoter regions. The avocado leaves in N3 have the highest activities of N-assimilating enzymes and N content as well as the lowest activities of antioxidant enzymes. Thus, 29.75 mg/L and 119.00 mg/L were chosen as low-N supply and normal-N supply for transcriptome analysis. The transcriptome analysis showed that *PaNRT1.11*, *PaNRT1.22*, *PaNRT1.32*, *PaNRT1.33*, *PaNRT1.38*, and *PaNRT1.52* and *PaNRT1.56* among *PaNRT1* members were up-regulated under normal-N condition in the leaves or roots, suggesting that these genes might affect N absorption under nitrate-sufficient conditions in avocado. RT-qPCR analysis found the relative expression patterns of selected genes among four samples were consistent with transcriptome data, suggesting that transcriptome data were reliable. **Conclusions:** This study would provide valuable information for identifying the functions of the *NRT* gene family in avocado.

## 1. Introduction

Nitrogen (N) is an essential mineral element involved in the plant growth and development process [1]. Insufficient N supply could inhibit photosynthesis as well as the synthesis of primary and secondary metabolites, consequently limiting plant growth and development [2]. Owing to low N availability in soil, N fertilizers have been widely applied to meet the N requirement of plant growth and development over the past half-century [3]. However, excessive use of N fertilizers, combined with N uptake and utilization by plants, leads to environmental pollution and increases production costs [4]. Therefore, it is essential to comprehensively understand the biological and molecular mechanism of N uptake and distribution to improve the efficiency of N uptake and use in plants.

Nitrate is the primary form of N taken by the higher plants [5], while nitrate reductase (NR), nitrite reductase (NiR), glutamine synthase (GS), glutamate synthase (GOGAT) play essential roles in N assimilation [6]. Superoxide dismutase (SOD), peroxidase (POD), and catalase (CAT). Inappropriate N supply could induce oxidative stress to inhibit plant growth, while the antioxidant enzymes can protect plant tissue by inhibiting oxidative stress [7]. In plants, high-affinity (HATS) and low-affinity nitrate-transport systems (LATS) were developed to cope with low- or high-nitrate environments [8]. Nitrate transporters (NRTs), including NRT1, NRT2, and NRT3 families, have been proven to regulate N uptake and transport in plants [9,10]. The NRT1 family, belonging to the peptide transporters (PTR) family of the Major Facilitator Super (MFS) family, is a low-affinity transporter [11]. 91 NRT1 members were found in rice, while *Arabidopis thaliana* consists of 53 NRT1 members, of which *AtNRT1.1-1.12* has been proven to be involved in nitrate transport [12]. As a high-affinity transporter, the NRT2 family belongs to the nitrate-nitrite-porter (NNP) family of the MFS family [13]. *MeNRT2.2* among six *NRT2* genes of *Manihot esculenta* was found to adapt to low N, while *AcNRT2.1* and *AcNRT2.2* among three *NRT2* genes of pineapple responded to nitrate starvation [9]. The NRT3 family serves HATS by interacting with the NRT2 family [11,14]. The interaction between *AtNRT3.1* and almost all members of the *AtNRT2* gene family in *Arabidopsis* has been observed, with the exception of *AtNRT2.7* [15]. Due to key *NRT* genes involved in N transport varying among various plants, clarifying the information on *NRT* genes is essential to improve N uptake and use in plants.

Avocado (*Persea americana* Mill.), a woody fruit tree of the Lauraceae family, is widely distributed in the tropical and subtropical regions of the world [16]. Owing to the high nutritional value and economic benefit of fruit, the worldwide fruit production and cultivation of avocado in 2022 exceeded 8.9 million tons and 884 thousand hectares, respectively [17]. Due to a lot of N being taken away with harvesting avocado fruit, avocado orchards require a large amount of N fertilizer to make up for the lost N each year [18]. Previous studies showed that long-term application of large amounts of N fertilizer resulted in loss of productivity, nitrogen loss, and groundwater pollution in avocado orchards [16,18,19]. Thus, clarifying the molecular mechanisms of N uptake and use is essential for achieving higher growth by reducing N supply in avocado. Although the chromosomal-level genome of avocado was published [20], the information on *PaNRT* genes has not yet been reported.

Thus, this study aimed to identify *PaNRT* genes, explore the chromosomal location, genetic structure, and phylogenetic relationship of the *PaNRT* gene family, and to investigate the expression profiles of *PaNRT* genes under low and normal N supply. These findings would facilitate clarification of the molecular regulatory network of N uptake and transportation in avocado.

## 2. Materials and Methods

### 2.1. Plant Materials

The experiment was conducted in the growth chamber under 25 °C/20 °C day/night cycle with a 14 h photoperiod. Seeds of avocado (cv. ‘Hass’) were germinated on non-woven with medium (coconut husk: peat soil: yellow soil = 4:4:2). Four months later, 54 uniform and healthy seedlings (seedling height approximately 35 cm and ground diameter approximately 4 mm) were selected and transferred to polypropylene containers (diameter and height are 10.6 cm and 9.5 cm, respectively) with coconut husk. 5 N supply concentrations were setup, including N1 (29.75 mg/L N), N2 (59.50 mg/L N), N3 (119.00 mg/L N), N4 (178.50 mg/L N), and N5 (238.00 mg/L N) (Table 1). N was supplied with the form of Ca(NO_3_)_2_·4H_2_O, KNO_3_ and NH_4_SO_4_. The concentrations of other elements were maintained at consistent levels in both the HN and LN treatments. Each treatment had three replicates and 3 seedlings for each replicate. 100 mL nutrient solutions (pH = 6.0, adjusted using NaOH and H_2_SO_4_) were added to the seedlings of the corresponding treatment. After 24 h, the leaves (on the third expanded leaf from the top) and roots for each treatment were collected and stored in liquid nitrogen. The leaves of 5 treatments were used to measure the activities of N-assimilating enzymes and antioxidant enzymes. Then, based on the activities of N-assimilating enzymes and antioxidant enzymes, the leaves and roots of low-N (LN) and normal-N treatments were chosen for transcriptome analysis.

### 2.2. Identification and Characterization of NRT Genes in Avocado

The sequence file of *Arabidopsis* NRT was retrieved from the TAIR database (https://www.arabidopsis.org/, accessed on 14 June 2024). The genome data of Avocado (GCA_029852735.1) was downloaded from the custom database. The protein sequence of *Arabidopsis* was blasted against the proteinic sequence of the avocado whole genome for obtaining potential *PaNRT* proteins by the BLASTP algorithm with an E-value < 0.01. To obtain the *PaNRT* domains, the potential *PaNRT* sequences were submitted to the Pfam (http://pfam.sanger.ac.uk/search, accessed on 14 June 2024) and SMART (http://smart.embl-heidelberg.de/, accessed on 14 June 2024). The reliable *PaNRT* proteins were retained by removing the sequences without the peptide transporter 2 (PTR2), Major Facilitator Super (MFS1), or nitrate assimilation related 2 (NAR2) domain. The ExPASy program (https://web.expasy.org/protparam/, accessed on 14 June 2024) was used to calculate the number of amino acids, the isoelectric point (pI), molecular weight (MW), instability index (II), and grand average of hydropathicity (GRAVY).

### 2.3. Chromosomal Localization and Phylogenetic Analysis of PaNRT Proteins

The chromosome localization data of *PaNRT* genes were obtained from the avocado genome database, and then the map of the chromosomal position of *PaNRT* genes and their relative distances was performed using the TBtools software (v2.0906) [21]. For the phylogenetic analysis, the full-length NRT amino acid sequences of *Arabidopsis* and avocado were aligned by Clustal W. MEGA 6 was employed to construct the phylogenetic tree using the neighbor-joining method with p-distance, pairwise deletion, and 1000 bootstrap replicates. Finally, the phylogenetic tree was visualized using Evolview (http://www.evolgenius.info/evolview/, accessed on 14 June 2024) [22].

### 2.4. Gene Structure, Conserved Motif, and Cis-Regulatory Element (CRE) Analysis of PaNRT Proteins

The gene structure of *PaNRTs* was analyzed using the web-based gene structure display server (http://gsds.cbi.pku.edu.cn/, accessed on 14 June 2024). MEME (ver. 5.1.1, http://meme-suite.org, accessed on 14 June 2024) was employed to analyze the conversed motifs of *PaNRT* proteins (the maximum motifs number of 10 and the motifs width of 6–50 aa). The 2000 bp upstream sequence of each gene was extracted and then loaded to the PlantCARE (http://bioinformatics.psb.ugent.be/webtools/plantcare/html/, accessed on 15 June 2024) to predict CRE. The TBtools software (v2.0906) was used to visualize this data information.

### 2.5. Measurement of the Physiological Indexes

The activities of N-assimilating enzymes (NR, NiR, GS, and GOGAT) and N content were measured following the method of Qin et al. [1]. The activities of antioxidant enzymes (Superoxide dismutase (SOD), peroxidase (POD), and catalase (CAT)) were performed using a kit (comin, Suzhou, China), and all enzyme activities were expressed as units per kilogram soluble protein [23].

### 2.6. RNA Sequencing and Gene Expression Analysis

Total RNA was extracted using an RNA Extraction Kit (Tiangen, Beijing, China), while the integrity, purity, and concentration of total RNA were checked using an agarose gel and NanoDrop^®^ ND-1000 portable UV-Vis Spectrophotometer (Thermo Scientific, Waltham, MA, USA). The Sequencing libraries were generated using NEBNext^®^Ultra™ RNA Library Prep Kit for Illumina^®^ (NEB, San Diego, CA, USA) following the manufacturer’s recommendations. Then, 12 cDNA libraries (including leaves and roots of N1 and N3) were sequenced using Illumina Hiseq 2000 platform and generating reads with a length of 2 × 100 bp. Raw data of fastq format were performed using in-house perl scripts. Raw sequences were transformed into clean reads after data processing. These clean reads were then mapped to the reference genome sequence of Avocado (GCA_029852735.1). The unigenes function was annotated based on Kyoto Encyclopedia of Genes and Genomes (KEGG) and Gene Ontology (GO) databases. RSEM was used to estimate gene expression levels [24]. Differentially expressed genes (DEG) were assigned using false discovery rate (FDR) < 0.01 and fold change (FC) ≥ 2 as thresholds. The enrichment of DEGs was analyzed based on GO (*p* value Cut off = 0.05, *p* Adjust Method = “BH”) and KEGG (*p* value Cut off = 0.05, *p* Adjust Method = “BH”, q value Cut off = 0.2) pathway analysis using the clusterProfiler R package with default parameters [25]. Based on the transcriptome data, TBtools software was employed to generate a heatmap of expression profiles of *PaNRT* genes.

RNA was reverse transcribed cDNA using a HiScript III 1st Strand cDNA Synthesis Kit (Vazyme, Nanjing, China). The real-time quantitative polymerase chain reaction (RT-qPCR) analysis was performed using Taq Pro Universal SYBR qPCR Master Mix (Vazyme, Nanjing, China) on the BioRad CFX96Real-Time PCR platform (BioRad, Hercules, CA, USA). The reaction system was 20 μL, including 10 μL Taq Pro Universal SYBR qPCR Master Mix, 2 μL cDNA, 0.4 μL each prime, and 7.2 μL ddH_2_O. The RT-qPCR program was as follow: 95 °C for 30 s, followed by 39 cycles 95 °C for 5 s, and 60 °C for 30 s; 95 °C for 15 s, 60 °C for 1 min, and 95 °C for 15 s. Three biological replicates were conducted, while 2^−∆∆CT^ method was used to calculate the relative expression levels with *PaActin* used as the internal control [26]. The primer for RT-qPCR is shown in Supplementary Table S1.

### 2.7. Statistical Analysis

One-way analysis of variance was performed to analyze the significance of difference (Duncan’s test, *p* < 0.05) using SPSS 19.0 statistical software (SPSS Inc, Chicago, IL, USA).

## 3. Results

### 3.1. Characterization of the PaNRT Gene Family

61 *NRT* genes were identified in the avocado genome using the *Arabidopsis NRTs* protein sequence as queries, including 57 *PaNRT1*, 3 *PaNRT2*, and 1 *PaNRT3* (Table 2). These *NRT* genes encoded from 389 (*PaNRT3*) to 1130 (*PaNRT1.19*) amino acids, while MW varied from 42.20 kDa (*PaNRT2.2*) to 125.09 kDa (*PaNRT1.19*). The pI of *PaNRT* proteins varied from 5.33 (*PaNRT1.29*) to 9.87 (*PaNRT2.2*), with 50 *PaNRTs* classified as alkaline proteins (pI > 7). The II of *PaNRT* proteins varied from 22.86 (*PaNRT1.53*) to 53.51 (*PaNRT1.12*), while 21 *PaNRTs* exhibited instability with II exceeding 40. The *PaNRT* proteins belong to hydrophobic proteins except *PaNRT3* (GRAVY < 0).

**Figure 1:**
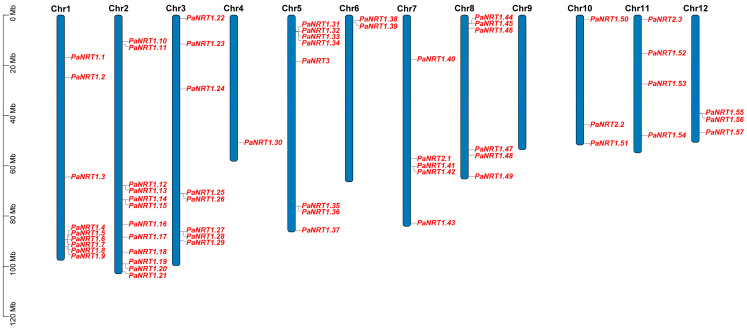
Distribution for the *PaNRT* genes on twelve chromosomes. The chromosome number is represented at each bar top. Mb, megabase.

In addition, the *NRT* genes (*PaNRT1.1*-*PaNRT1.57*/*PaNRT2.1*-*PaNRT2.3*/*PaNRT3*) were named based on the locations in the chromosomes (Figure 1). These *NRT* genes were mapped on 11 avocado chromosomes including Chr1 (9 *PaNRTs*), Chr2 (12 *PaNRTs*), Chr3 (8 *PaNRTs*), Chr4 (1 *PaNRTs*), Chr5 (8 *PaNRTs*), Chr6 (2 *PaNRTs*), Chr7 (5 *PaNRTs*), Chr8 (6 *PaNRTs*), Chr10 (3 *PaNRTs*), Chr11 (4 *PaNRTs*), and Chr12 (3 *PaNRTs*) (Table 2).

### 3.2. Phylogenetic Relationship and Conserved Motifs of PaNRTs

**Figure 2:**
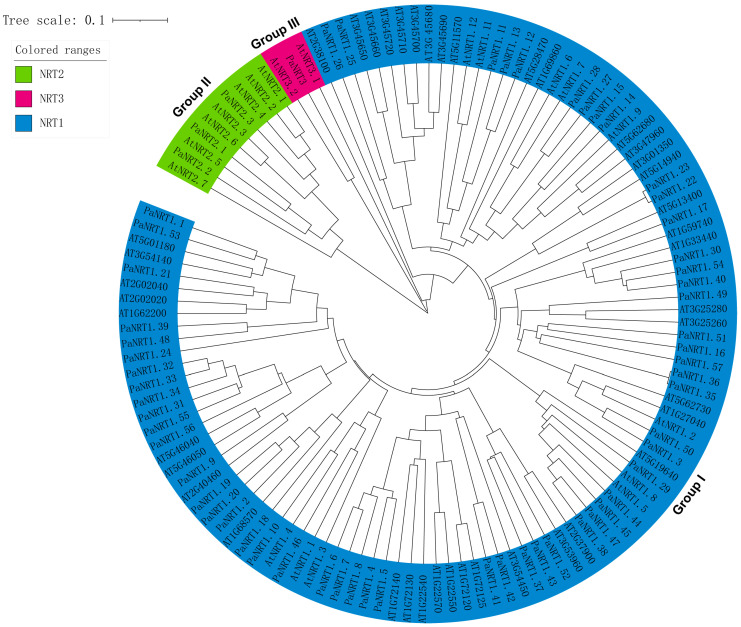
Phylogenetic analysis of NRT protein involved 62 *Arabidopsis* NRT protein and 61 avocado NRT protein sequences. The tree is further clustered into 3 subfamilies, which are shown in different colors.

A Neighbor-Joining phylogenetic tree was performed using the full-length amino acid sequences of *NRTs* from avocado and *Arabidopsis*, which reflected the evolutionary history and functional association of avocado *NRT* proteins (Figure 2). The 61 *PaNRTs* were clustered into three groups. 57 *PaNRT1* were clustered in Group Ⅰ, while 3 *PaNRT2* and 1 *PaNRT3* family were classified into Group Ⅱ and Group Ⅲ respectively.

**Figure 3:**
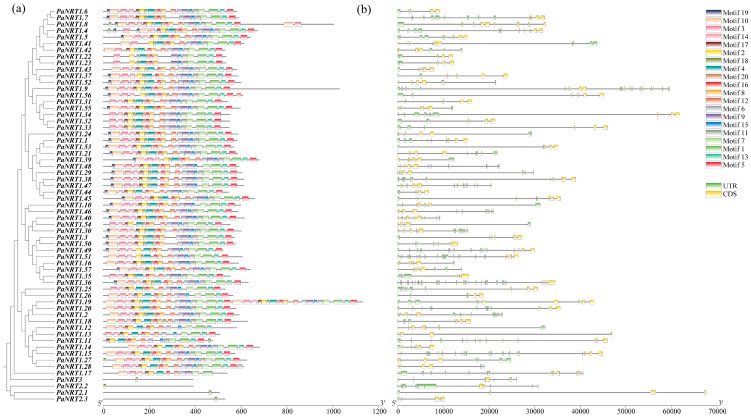
Phylogenetic relationships, motif distribution, and gene structure of *PaNRT* genes. (**a**) The phylogenetic relationships of 61 *PaNRT* proteins using the neighbor-joining method and the conserved domain architecture of the *PaNRT* proteins. (**b**) Gene structure analysis of the *PaNRT* gene family. The bars of yellow and blue represent UTR and CDS, respectively.

In addition, the relationships of the *PaNRTs* were exhibited deeply using phylogenetic analysis (Figure 3a). All *PaNRT1* members contain motif 1, motif 2, motif 4, motif 7, and motif 16, indicating that these might be the most conserved motifs in the NRT1 family. However, the members of *PaNRT2* and *PaNRT3* only contained motif 18. Subsequently, the gene structure of the *PaNRT* family was constructed to investigate the differences in structure (Figure 3b). The *PaNRT* family gene sequences exhibited significant variation in length, ranging from the longest DNA sequence (*PaNRT1.44*) at nearly 70 kb to the shortest sequence (*PaNRT1.9*) at less than 10 kb. The number distribution of exon-intron structure of the *PaNRT* family showed an obvious difference, with the number of intron and exon varied 1–9 and 2–10, respectively.

### 3.3. Characterization of CREs in the Promoter Regions of PaNRTs

**Figure 4:**
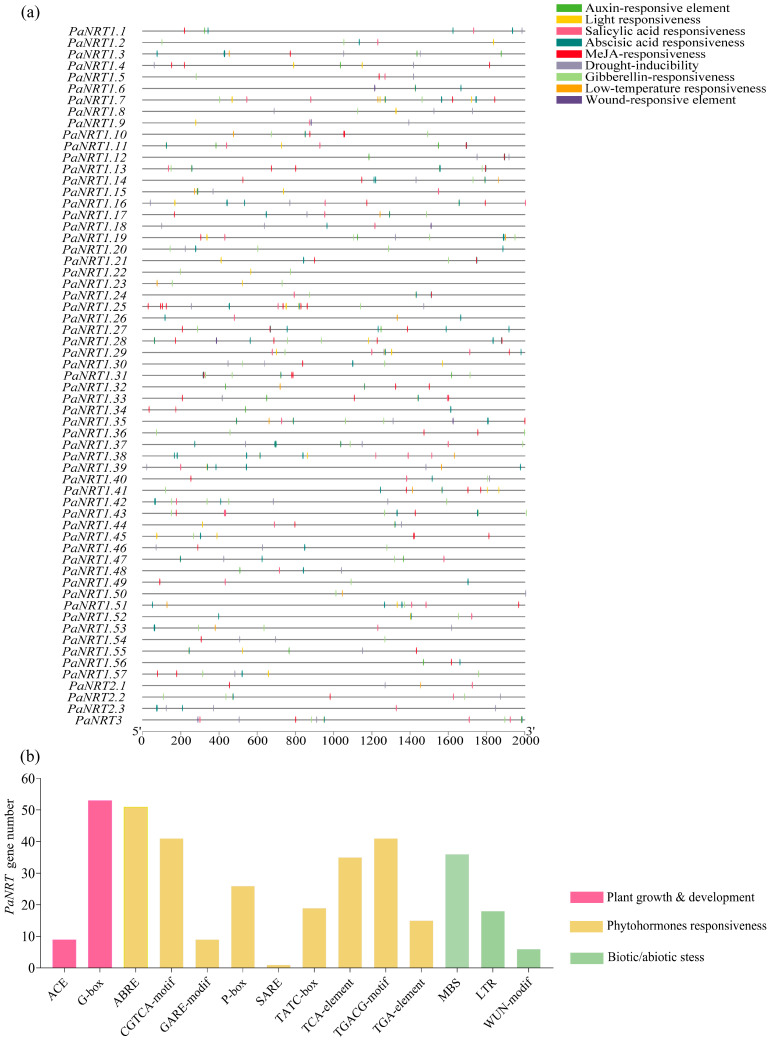
Characterization of *cis*-elements in the promoter regions of *PaNRT* genes. (**a**) Distribution of *cis*-elements in different colored rectangles. The y-axis indicates the upstream length to the translation start site. (**b**) The number of *PaNRT* genes harboring different *cis*-elements.

As shown in Figure 4a and Table S2, the characterization of CREs in the promoter regions of *PaNRTs* was analyzed. In the process of plant growth and development, phytohormones response, and stress response, some vital CREs were found in the promoter regions of *PaNRTs*, including auxin-responsive element, light responsiveness, salicylic acid responsiveness, abscisic acid responsiveness, MeJA-responsiveness, drought-inducibility, gibberellin-responsiveness, low-temperature responsiveness, and wound-responsive element. 54 *PaNRT* genes contained light responsiveness, including ACE and G-Box. Abscisic acid responsiveness (ABRE) was found in the promoter regions of 51 *PaNRT* genes, while MeJA-responsiveness (CGTCA and TGACG motif) was distributed in 41 *PaNRT* genes. Gibberellin-responsiveness (P-box, TATC-box, and GARE-motif), drought-inducibility (MBS), and salicylic acid responsiveness were identified in 39, 36, and 35 *PaNRT* genes, respectively. In addition, approximately 29.6%, 24.6%, and 9.8% of *PaNRT* genes contained low-temperature responsiveness (LTR), auxin-responsive element (TGA-element), and wound-responsive element (WUN motif), respectively (Figure 4b).

### 3.4. Physiological Indexes in Avocado Leaves Under Different N Supply

As shown in Table 3, N supply significantly affected the activities of N-assimilating enzymes (NR, NiR, GS, and GOGAT) in the leaves of avocado (*p* < 0.05). As the N supply increased, the activities of NR, NiR, GS, and GOGAT showed an unimodal pattern, peaking at N3. Compared to N3, the activities of NR, NIR, GS, and GOGAT in avocado leaves decreased by 66.29%, 71.91%, 61.73%, and 70.59% in N1 treatment, 32.41%, 46.90%, 28.72%, and 38.66% in N2 treatment, 43.08%, 53.06%, 33.22%, and 44.54% in N4 treatment, and 64.35%, 77.86%, 68.72%, and 78.15% in N5 treatment.

In addition, significant differences were observed in the activities of antioxidant enzymes (SOD, POD, and CAT) among N supply (Table 3, *p* < 0.05). The activities of SOD, POD, and CAT were all in the order of N5 > N1 >N4 > N2 > N3. Compared to N3, the activities of antioxidant enzymes improved by 250.05–294.00% in N1 treatment, 106.72–137.00% in N2 treatment, 132.60–180.00% in N4 treatment, and 313.74–402.00% in N5 treatment. The highest N content of leaves was achieved in N3 (Table 3, *p* < 0.05). Compares to N3, the N content in N1, N2, N4, and N5 reduced by 39.73%, 24.80%, 32.62%, and 47.29%, respectively.

### 3.5. RNA-Seq Analysis in Avocado Under Different N Supply

Based on the activities of N-assimilating enzymes and antioxidant enzymes in avocado leaves, N1 and N2 had low N levels for the growth of avocado seedlings, while N3 had a normal N level. The leaves and roots of N1 and N3 were used as low-N supply and normal-N supply for transcriptome analysis. In total, 72.97 gigabytes (Gb) of clean data from 12 samples were obtained. The GC% of the clean data ranged from 44.73% to 46.53%, and the percentage of Q30 from all the samples ranged from 96.21% to 96.93%, indicating that the quality and accuracy of sequencing data were sufficient for further analysis.

The mapped ratio of clean reads in each sample to the assembled Transcript or Unigene library ranged from 93.22% to 96.16%. Then, only mapped reads were used in the subsequent analysis. Fragments Per Kilobase of transcript per Million mapped reads (FPKM) values were used to represent the abundance of each unigene. Moreover, Pearson correlation analysis for estimated gene expression levels showed that a strong correlation (R^2^ > 0.84) existed between the replicated of each treatment (Figure S1a). On the contrary, a weaker correlation was observed between different samples. As illustrated in Figure S1b, the PCA plot indicated a little intragroup variation for QC samples.

**Figure 5:**
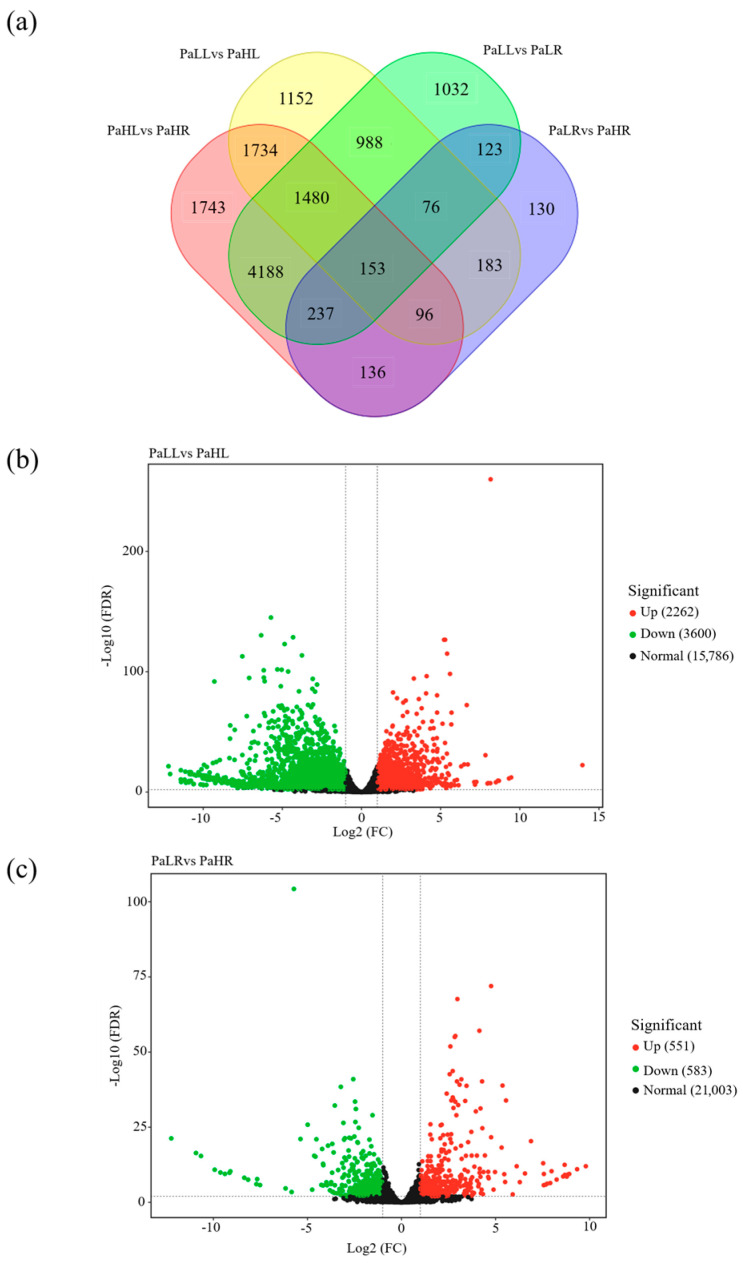
Differentially expressed genes (DEGs) of leaf and root in avocado under different N supply conditions. (**a**) The display of DEGs in PaLL, PaLR, PaHL, and PaHR. (**b**) The up-regulated and downregulated DEGs in PaLL vs. PaHL group. (**c**) The up-regulated and downregulated DEGs in PaLR vs. PaHR group.

5862 DEGs were identified between PaLL (leaf under low-N supply) and PaHL (leaf under normal-N supply), in which 2262 DEGs were up-regulated and 3600 DEGs were down-regulated (Figure 5a,b). The comparison between PaLR (root under low N supply) and PaHR (root under normal-N supply) resulted in 551 up-regulated and 583 down-regulated DEGs (Figure 5a,c). For DEGs of the PaLL vs. PaHL and PaLR vs. PaHR groups, the metabolic process, cellular process, single-organism process, membrane, cell, cell part, membrane part, catalytic activity, and blinding were both the most enriched categories in GO enrichment analysis (Figure S2). In KEGG enrichment analysis, DEGs of the PaLL vs. PaHL group were mainly involved in ribosome, plant hormone signal transduction, phenylpropanoid biosynthesis, and plant-pathogen interaction, while DEGs of the PaLR vs. PaHR group were responsible for plant hormone signal transduction, plant-pathogen interaction, phenylpropanoid biosynthesis, carbon metabolism, and protein processing in endoplasmic reticulum (Figure S3).

### 3.6. Expression Profiles of PaNRT Genes Under Different N Supply

**Figure 6:**
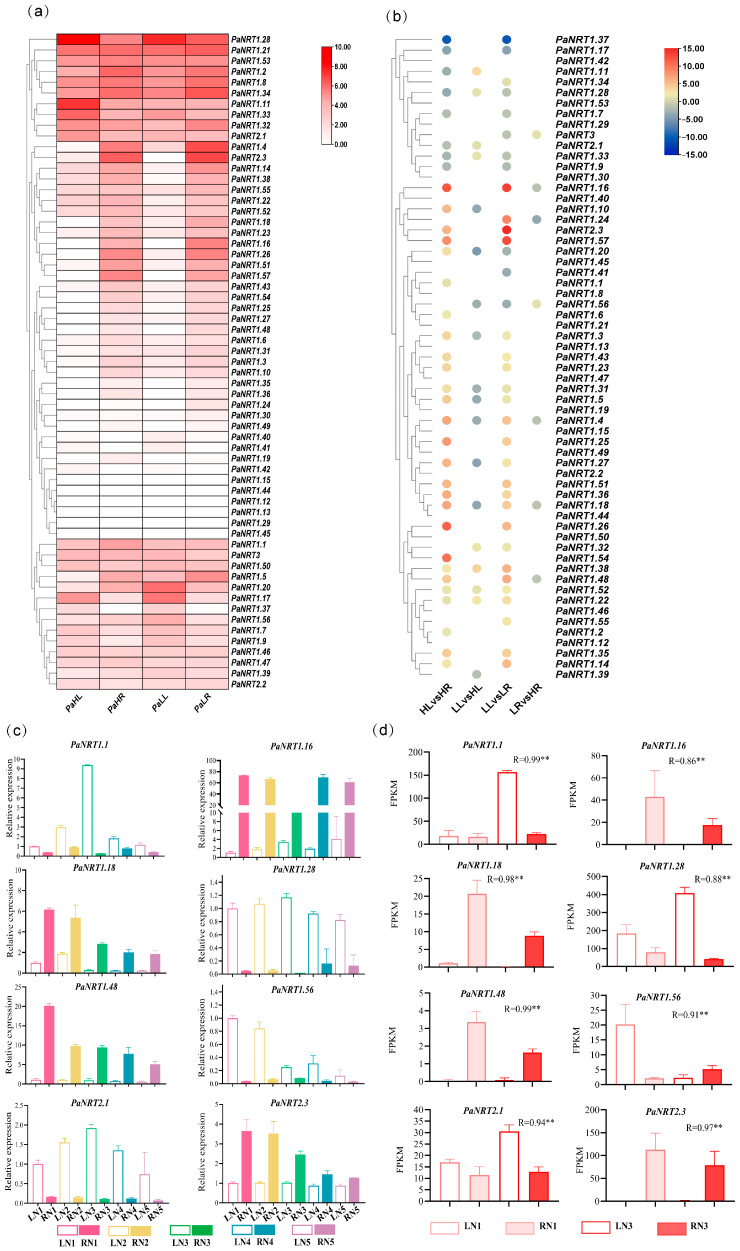
The expression profiles of *PaNRT* genes in the leaf and root under different N conditions. (**a**) Heatmap illustrates the expression levels of *PaNRT* genes. (**b**) Fold change of *PaNRT* genes. (**c**) Expression data from RNA-Seq by RT-qPCR. (**d**) Validation of 8 *PaNRT* genes’ FPKM. Different letters indicate significant differences among different samples (*p* < 0.05). R value indicates Pearson correlation coefficients between RNA-seq and RT-qPCR data, ** indicates a significance at *p* < 0.05.

Transcriptome data was used to analyze the expression profiles of *PaNRT* genes to explore their potential role and tissue-specific characteristics under normal-N and low-N supply. Figure 6a,b showed that no expression (FPKM < 1) was found in 11 *PaNRTs* genes of four samples. *PaNRT1.6*, *PaNRT1.16*, *PaNRT1.19*, *PaNRT1.25*, *PaNRT1.26*, *PaNRT1.27*, *PaNRT1.35*, *PaNRT1.36*, *PaNRT1.43*, *PaNRT1.48*, *PaNRT1.51*, *PaNRT1.54*, and *PaNRT1.57* were mainly expressed in the root, while only one gene (*PaNRT1.37*) was mainly expressed in the leaf. Compared to low-N supply in the leaf, *PaNRT1.11*, *PaNRT1.22*, *PaNRT1.32*, *PaNRT1.33*, *PaNRT1.38*, *PaNRT1.52*, and *PaNRT2.1* were highly expressed under normal-N supply, while *PaNRT1.3*, *PaNRT1.4*, *PaNRT1.5*, *PaNRT1.10*, *PaNRT1.18*, *PaNRT1.31*, *PaNRT1.39*, and *PaNRT1.56* were lowly expressed. Compared to low-N supply, the expressions of *PaNRT3* and *PaNRT1.56* in the root were highly expressed, while the expression levels of *PaNRT1.4*, *PaNRT1.16*, *PaNRT1.18*, *PaNRT1.24*, and *PaNRT1.48* were down-regulated. To verify the reliability of transcriptome data, eight *PaNRT* genes (*PaNRT1.11*, *PaNRT1.16*, *PaNRT1.18*, *PaNRT1.28*, *PaNRT1.48*, *PaNRT1.56*, *PaNRT2.1*, and *PaNRT2.3*) were selected for RT-qPCR analysis (Figure 6c). Pearson correlation analysis showed that the relative expression of eight *PaNRT* genes was positively associated with FPKM values (Figure 6).

## 4. Discussion

Nitrate is one of the primary N forms absorbed by plants, while NRT genes are responsible for the uptake and transportation of nitrate in plants [27]. The critical roles of NRT genes have been identified in some woody plants, such as *Eucalyptus grandis* [21], apple [27], and poplar [28]. However, the identification and functional roles of the *PaNRT* gene family have not been characterized until now. In the present work, a total of 61 *PaNRTs* were identified at the genome level in avocado (Table 2). In addition, gene structure, phylogenetic relationship, and expression profiles of these *PaNRT* genes were characterized (Table 2). To the best of our knowledge, this study represents the first comprehensive report on the characterization of the *NRT* gene family in avocado. Compared to *Arabidopsis*, the number of *PaNRT* genes slightly decreased. However, the number of NRT family members varied greatly among species, such as 75 members in *E. grandis* [21], 39 members in radish [2], and 84 members in apple [27]. The number of *NRT* genes in avocado was less than in apple and *E. grandis*, suggesting the contraction of the NRT family during evolution. The species-specific adaptations may be the reason for this contraction [29], while avocado might not require as many *NRT* genes for N transport.

An uneven pattern was found in the distribution of *PaNRT* genes in 11 avocado chromosomes (Figure 1), supported by other reports on *E. grandis* [21] and apple [27]. This result suggested that the chromosomes with relatively more genes (like Chr 2) have undergone the duplication event or contributed more to N transport [27]. The structure (the number of intron and exon as well as the number and order of motifs) varied among *NRT* genes in avocados (Figure 3), consistent with the reports from radish [2] and *E. grandis* [21]. The structure difference may affect the transport efficiency of *NRT* genes [29]. In a word, understanding the distribution pattern of chromosomes and gene structure would contribute to identifying genomic regions and genes involved in the N transport of avocado.

CREs in the *PaNRTs* promoter were associated with phytohormonal response and various stresses (including light, drought, low temperature, and wound) (Figure 4). This finding was consistent with the results from radish [2] and *E. grandis* [21], indicating that *PaNRT* genes play an essential role in regulating the growth, development, and stress adaption of plants. Interestingly, the *PaNRTs* promoter found many CREs related to the phytohormonal response (Figure 4 and Table S2). Moreover, KEGG analysis confirmed that DEGs in avocado under different N conditions were associated with plant hormone signal transduction (Figure S2). Other plants also reported that *NRT* gene expression can be modulated by phytohormone signaling, while phytohormone biosynthesis and transportation (such as gibberellin, salicylic acid, and abscisic acid) can be regulated by *NRT* genes [30,31,32,33]. However, as shown in GO and KEGG analysis (Figures S2 and S3), the regulatory mechanisms of *PaNRTs* expression are complex and need more research work.

N-assimilating enzymes are essential for N uptake in plants, which is influenced by N additions [31]. The N content and activities of N-assimilating enzymes in N3 were significantly higher than those of other treatments (Table 3), suggesting that 119.00 mg/L was the optimal N supply for avocado seedling growth in this study. In addition, the antioxidant enzymes can mitigate damage of plant tissue in low-N and high-N stresses [8]. Our results showed that the activities of SOD, POD, and CAT were the lowest in N3 (Table 3), supported the above conclusion. Thus, N1 and N3 were chosen as low-N supply and normal-N supply of avocado seedlings growth for transcriptome analysis. Previous studies showed that NRT gene expressions differed concerning the tissue [28,34]. Based on transcriptome data, this study chose eight genes among DEGs of tissue-specific under different N conditions. The relative expression patterns of these genes among four samples were consistent with transcriptome data (Figure 6), suggesting that the expression profiles of transcriptome data were reliable. Here, the transcriptome and RT-qPCR analysis demonstrated that the *PaNRT* genes exhibited leaf- or root-specific expression patterns (Figure 6). In addition, the NRT family members play diverse roles in the absorption and allocation of nitrate [35]. The subfamily NRT1 belonging to LATS proteins was highly expressed in the normal nitrate environment, while NRT2 and NRT3 subfamilies belonging to LATS proteins were opposite [21]. In this study, *PaNRT1.11*, *PaNRT1.22*, *PaNRT1.32*, *PaNRT1.33*, *PaNRT1.38*, and *PaNRT1.52*, and *PaNRT1.56* among *PaNRT1* members were up-regulated under normal-N conditions in the leaves or roots, while the *NRT2* and *NRT3* genes did not show high expressions either in the leaves or in the roots at the low N supply (Figure 6a,b). These results suggested that *PaNRT1.11*, *PaNRT1.22*, *PaNRT1.32*, *PaNRT1.33*, *PaNRT1.38*, *PaNRT1.52*, and *PaNRT1.56* might be involved in N absorption under nitrate-sufficient conditions in avocado (Figure 6c). Further studies are necessary to determine the specific role of these *PaNRT* genes in avocado N absorption.

## 5. Conclusions

In this study, 61 *NRT* genes were identified in avocado. Many cis-regulatory elements in the *PaNRTs* promoter were associated with phytohormonal response and various stresses. Expression pattern analysis showed that *PaNRT1.11*, *PaNRT1.22*, *PaNRT1.32*, *PaNRT1.33*, *PaNRT1.38*, and *PaNRT1.52*, and *PaNRT1.56* were significantly up-regulated under normal-N conditions in both leaves or roots, indicating that these genes are crucial for N absorption in avocado. This study would provide valuable information for identifying the functions of *NRT* gene family in avocado. In addition, it contributes to the breeding of avocado varieties with high N uptake and utilization for reducing the use and loss of N fertilizer. 

## Figures and Tables

**Table 1:** Formulation of nutrient solutions with different nitrogen concentrations.

Nutrient Elements	N1 (mg/L)	N2 (mg/L)	N3 (mg/L)	N4 (mg/L)	N5 (mg/L)
N	29.75	59.50	119.00	178.50	238.00
P	15.50	15.50	15.50	15.50	15.50
K	298.00	298.00	298.00	298.00	298.00
Ca	210.00	210.00	210.00	210.00	210.00
Mg	48.10	48.10	48.10	48.10	48.10
B	0.50	0.50	0.50	0.50	0.50
Mn	0.50	0.50	0.50	0.50	0.50
Zn	0.50	0.50	0.50	0.50	0.50
Cu	0.50	0.50	0.50	0.50	0.50
Mo	0.05	0.05	0.05	0.05	0.05
Fe	5.60	5.60	5.60	5.60	5.60
Cl	553.45	519.72	395.47	271.22	146.74
Na	4.60	4.60	4.60	4.60	4.60

**Table 2:** Characterization of *PaNRT* family genes in avocado.

Gene Name	Gene ID	Chromosome	Amino Acid Number (aa)	Isoelectric Point (pI)	Molecular Weight (Mw, kDa)	Instability Index (II)	Grand Average of Hydropathicity (GRAVY)
*Pa* *NRT1.1*	KAJ8648131.1	Chr1	582	8.55	65.66	28.62	0.071
*PaNRT1.2*	KAJ8648506.1	Chr1	591	6.90	65.80	34.01	0.163
*PaNRT1.3*	KAJ8649386.1	Chr1	570	8.26	62.41	47.64	0.451
*PaNRT1.4*	KAJ8650603.1	Chr1	673	8.84	73.85	42.48	0.237
*PaNRT1.5*	KAJ8650604.1	Chr1	641	8.99	70.60	42.03	0.262
*PaNRT1.6*	KAJ8650605.1	Chr1	581	6.82	64.02	41.71	0.386
*PaNRT1.7*	KAJ8650614.1	Chr1	591	6.66	65.06	41.51	0.351
*PaNRT1.8*	KAJ8650615.1	Chr1	1003	8.83	109.84	39.17	0.059
*PaNRT1.9*	KAJ8650804.1	Chr1	1028	8.82	115.40	38.84	0.065
*PaNRT1.10*	KAJ8643124.1	Chr2	599	8.75	65.72	32.66	0.403
*PaNRT1.11*	KAJ8643206.1	Chr2	476	8.98	52.64	35.40	0.190
*PaNRT1.12*	KAJ8644789.1	Chr2	581	9.02	63.81	53.51	0.289
*PaNRT1.13*	KAJ8644791.1	Chr2	507	8.76	55.89	40.64	0.363
*PaNRT1.14*	KAJ8645062.1	Chr2	680	8.82	75.05	36.46	0.185
*PaNRT1.15*	KAJ8645064.1	Chr2	574	8.86	63.43	33.50	0.369
*PaNRT1.16*	KAJ8645606.1	Chr2	589	8.56	64.59	33.15	0.351
*PaNRT1.17*	KAJ8645953.1	Chr2	539	7.14	59.38	35.94	0.178
*PaNRT1.18*	KAJ8646339.1	Chr2	628	8.58	69.00	36.58	0.164
*PaNRT1.19*	KAJ8646648.1	Chr2	1130	8.73	125.09	37.08	0.283
*PaNRT1.20*	KAJ8646649.1	Chr2	578	8.82	63.82	36.49	0.265
*PaNRT1.21*	KAJ8646894.1	Chr2	585	5.55	64.36	27.09	0.333
*PaNRT1.22*	KAJ8634464.1	Chr3	534	8.83	59.55	38.84	0.336
*PaNRT1.23*	KAJ8635043.1	Chr3	534	8.93	59.65	41.24	0.306
*PaNRT1.24*	KAJ8635775.1	Chr3	586	8.92	64.87	25.21	0.293
*PaNRT1.25*	KAJ8637006.1	Chr3	532	9.06	57.82	34.75	0.425
*PaNRT1.26*	KAJ8637010.1	Chr3	564	9.11	61.16	37.97	0.436
*PaNRT1.27*	KAJ8637866.1	Chr3	625	8.64	69.15	40.26	0.153
*PaNRT1.28*	KAJ8637867.1	Chr3	611	9.10	67.60	40.54	0.233
*PaNRT1.29*	KAJ8638117.1	Chr3	604	5.33	66.19	23.04	0.376
*PaNRT1.30*	KAJ8618733.1	Chr4	602	9.01	66.20	44.95	0.320
*PaNRT1.31*	KAJ8639158.1	Chr5	543	9.47	60.90	33.41	0.294
*PaNRT1.32*	KAJ8639160.1	Chr5	550	9.13	61.89	36.91	0.305
*PaNRT1.33*	KAJ8639166.1	Chr5	548	9.22	61.49	36.79	0.335
*PaNRT1.34*	KAJ8639169.1	Chr5	551	9.32	61.53	30.50	0.305
*PaNRT3*	KAJ8639622.1	Chr5	389	6.48	43.81	49.26	-0.398
*PaNRT1.35*	KAJ8641744.1	Chr5	552	8.98	61.36	35.84	0.292
*PaNRT1.36*	KAJ8641745.1	Chr5	631	8.90	69.79	35.37	0.178
*PaNRT1.37*	KAJ8642414.1	Chr5	587	9.21	66.10	43.17	0.224
*PaNRT1.38*	KAJ8625967.1	Chr6	613	6.76	68.27	28.61	0.165
*PaNRT1.39*	KAJ8626045.1	Chr6	679	6.69	75.60	33.93	0.199
*PaNRT1.40*	KAJ8629498.1	Chr7	615	9.05	68.12	39.34	0.276
*PaNRT2.1*	KAJ8630468.1	Chr7	506	9.05	54.10	39.77	0.381
*PaNRT1.41*	KAJ8630599.1	Chr7	613	8.30	67.57	48.46	0.335
*PaNRT1.42*	KAJ8630600.1	Chr7	531	8.90	58.82	41.00	0.460
*PaNRT1.43*	KAJ8631817.1	Chr7	582	8.50	65.23	41.23	0.161
*PaNRT1.44*	KAJ8632061.1	Chr8	546	6.57	60.94	32.89	0.139
*PaNRT1.45*	KAJ8632063.1	Chr8	659	6.79	73.01	28.70	0.213
*PaNRT1.46*	KAJ8632194.1	Chr8	586	9.04	64.78	34.61	0.288
*PaNRT1.47*	KAJ8633780.1	Chr8	612	7.82	68.15	33.15	0.143
*PaNRT1.48*	KAJ8633886.1	Chr8	590	6.37	65.42	33.31	0.301
*PaNRT1.49*	KAJ8634373.1	Chr8	521	9.11	58.11	41.06	0.362
*PaNRT1.50*	KAJ8621555.1	Chr10	575	9.25	63.17	49.25	0.410
*PaNRT2.2*	KAJ8623074.1	Chr10	393	9.87	42.20	37.24	0.585
*PaNRT1.51*	KAJ8623589.1	Chr10	605	8.54	67.25	24.56	0.285
*PaNRT2.3*	KAJ8623672.1	Chr11	530	9.01	57.24	42.03	0.335
*PaNRT1.52*	KAJ8624254.1	Chr11	599	9.04	67.00	41.35	0.208
*PaNRT1.53*	KAJ8624412.1	Chr11	569	8.69	63.58	22.86	0.272
*PaNRT1.54*	KAJ8625368.1	Chr11	552	8.89	61.29	42.34	0.326
*PaNRT1. 55*	KAJ8616190.1	Chr11	597	9.39	67.05	38.00	0.252
*PaNRT1. 56*	KAJ8616201.1	Chr11	607	8.42	68.21	27.41	0.159
*PaNRT1. 57*	KAJ8616588.1	Chr11	640	9.12	71.34	30.74	0.236

**Table 3:** Effects of N supply on physiological indexes in avocado leaves.

Treatment	Nitrate Reductase (NR) Activity (µg/(h^2^·g FW))	Nitrite Reductases (NiR) Activity (µg/(h^2^·g FW))	Glutamine Synthease (GS) Activity (µg/(h^2^·g FW))	Glutamate Synthase (GOGAT) Activity (U/mg FW)	Superoxide Dismutase (SOD) Activity (U/g FW)	Peroxidase (POD) Activity (U/g FW)	Catalase (CAT) Activity (U/g FW)	Nitrogen Content (mg/g)
N1	11.47 ± 1.14 c	48.13 ± 3.73 c	16.43 ± 1.01 c	0.35 ± 0.04 c	34.90 ± 2.65 a	3.94 ± 0.35 b	0.39 ± 0.03 a	6.78 ± 0.68 c
N2	23.00 ± 1.71 b	90.97 ± 11.37 b	30.60 ± 2.44 b	0.73 ± 0.06 b	20.61 ± 1.54 b	2.37 ± 0.19 c	0.23 ± 0.03 b	8.46 ± 0.78 b
N3	34.03 ± 1.86 a	171.33 ± 14.90 a	42.93 ± 3.03 a	1.19 ± 0.08 a	9.97 ± 0.76 c	1.00 ± 0.09 d	0.11 ± 0.01 c	11.25 ± 0.71 a
N4	19.37 ± 1.40 b	80.43 ± 7.18 b	28.67 ± 2.48 b	0.66 ± 0.09 b	23.19 ± 2.14 b	2.80 ± 0.19 c	0.29 ± 0.03 b	7.58 ± 0.44 b
N5	12.13 ± 0.83 c	37.93 ± 4.93 c	13.43 ± 1.01 c	0.26 ± 0.03 c	41.25 ± 2.98 a	5.02 ± 0.42 a	0.46 ± 0.04 a	5.93 ± 0.42 c

Notes: Different letters indicate significant differences among different samples (*p* < 0.05).

## Data Availability

The data reported in this paper were deposited in the Genome Sequence Archive (Genomics, Proteomics, and Bioinformatics 2021) at the National Genomics Data Center, China National Center for Bioinformation/Beijing Institute of Genomics, Chinese Academy of Sciences (Bioproject: PRJCA029897), and are publicly accessible at https://ngdc.cncb.ac.cn/gsa, accessed on 5 September 2024.

## Supplementary Materials

The following supporting information can be downloaded at: https://www.mdpi.com/article/10.3390/genes15121600/s1, Figure S1: Correlation analysis (a) and principal component analysis (PCA) (b) of the samples in transcriptome results; Figure S2: GO analysis of DEGs in PaLL vs. PaHL and PaLR vs. PaHR groups; Figure S3: KEGG-enrichment analysis of DEGs in PaLL vs. PaHL and PaLR vs. PaHR groups; Table S1: Primers used in this study; Table S2: The potential *cis*-elements in the promoter region of 61 *PaNRT* genes; Table S3: Statistics of the RNA-seq data; Table S4: The annotation rate of avocado genes in 8 different databases.
