# Supplementary figures and images for: Genome-Wide Identification and Expression Analysis of PaNRT Gene Family Under Various Nitrogen Conditions in Avocado (Persea americana Mill.)
Source: Genes (Basel). 2024 Dec 14;15(12):1600. doi: 10.3390/genes15121600 (PMC11675230; doi:10.3390/genes15121600)

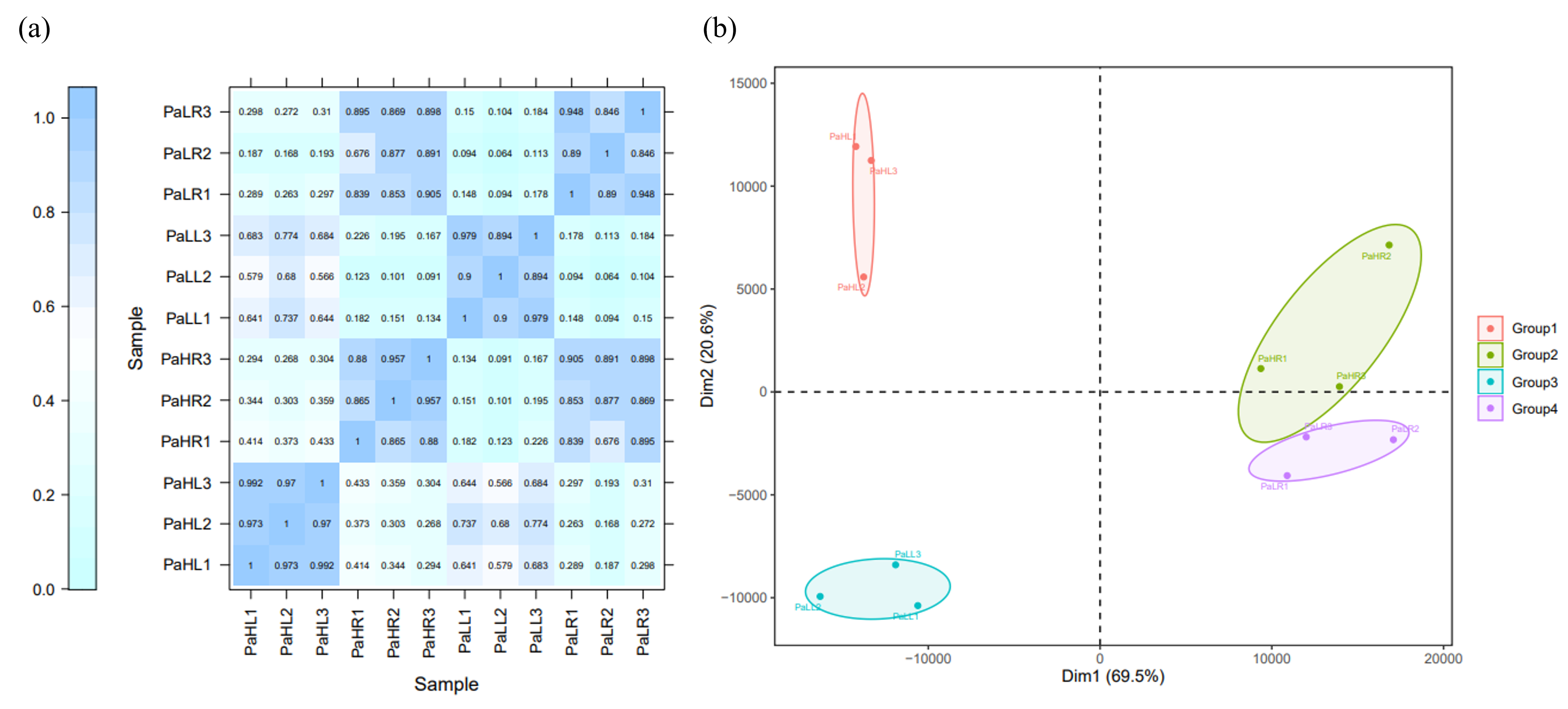

Supplement: Supplementary file 1 [file genes-15-01600-s001.zip › Figure S1.tif]

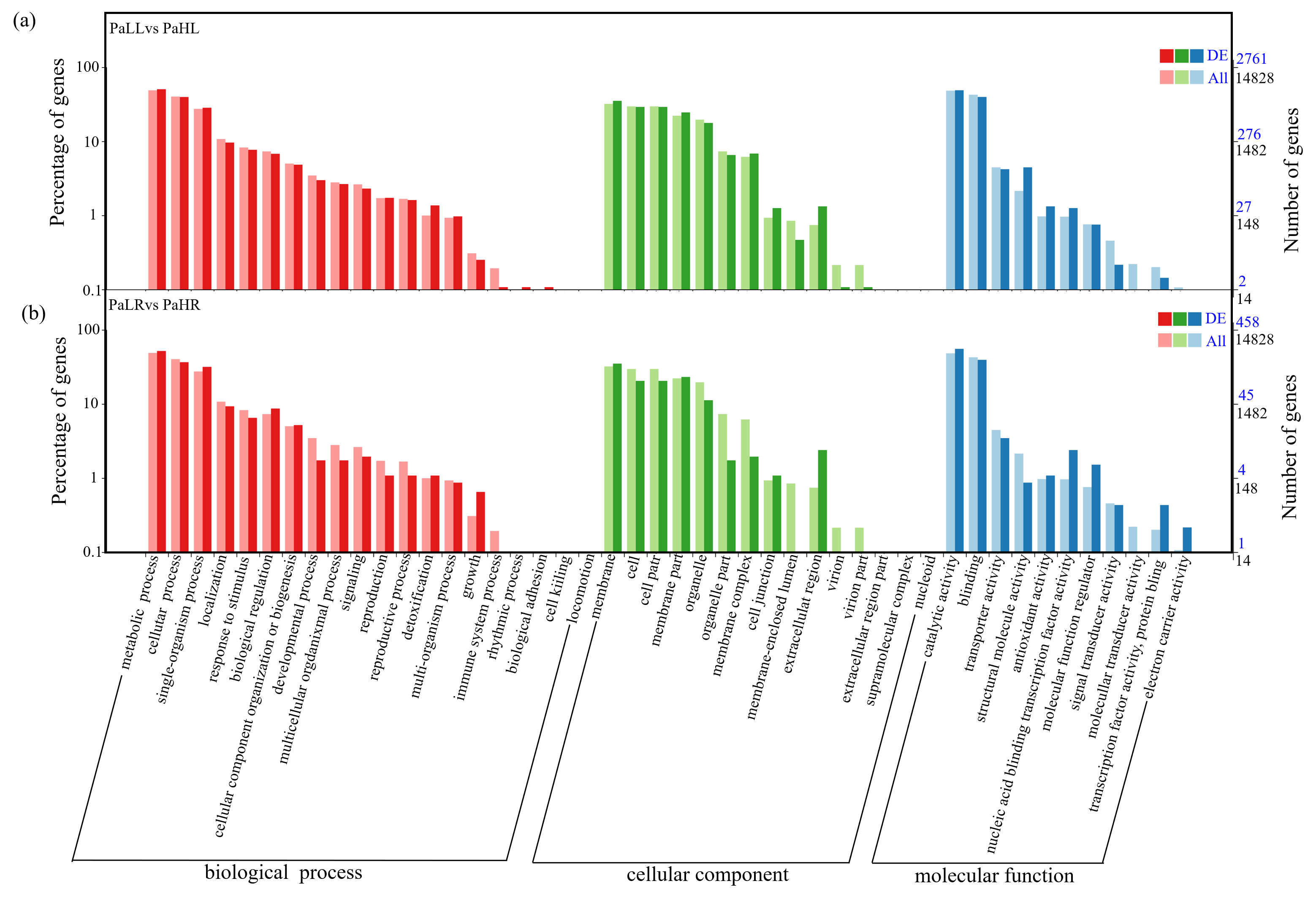

Supplement: Supplementary file 1 [file genes-15-01600-s001.zip › Figure S2.tif]

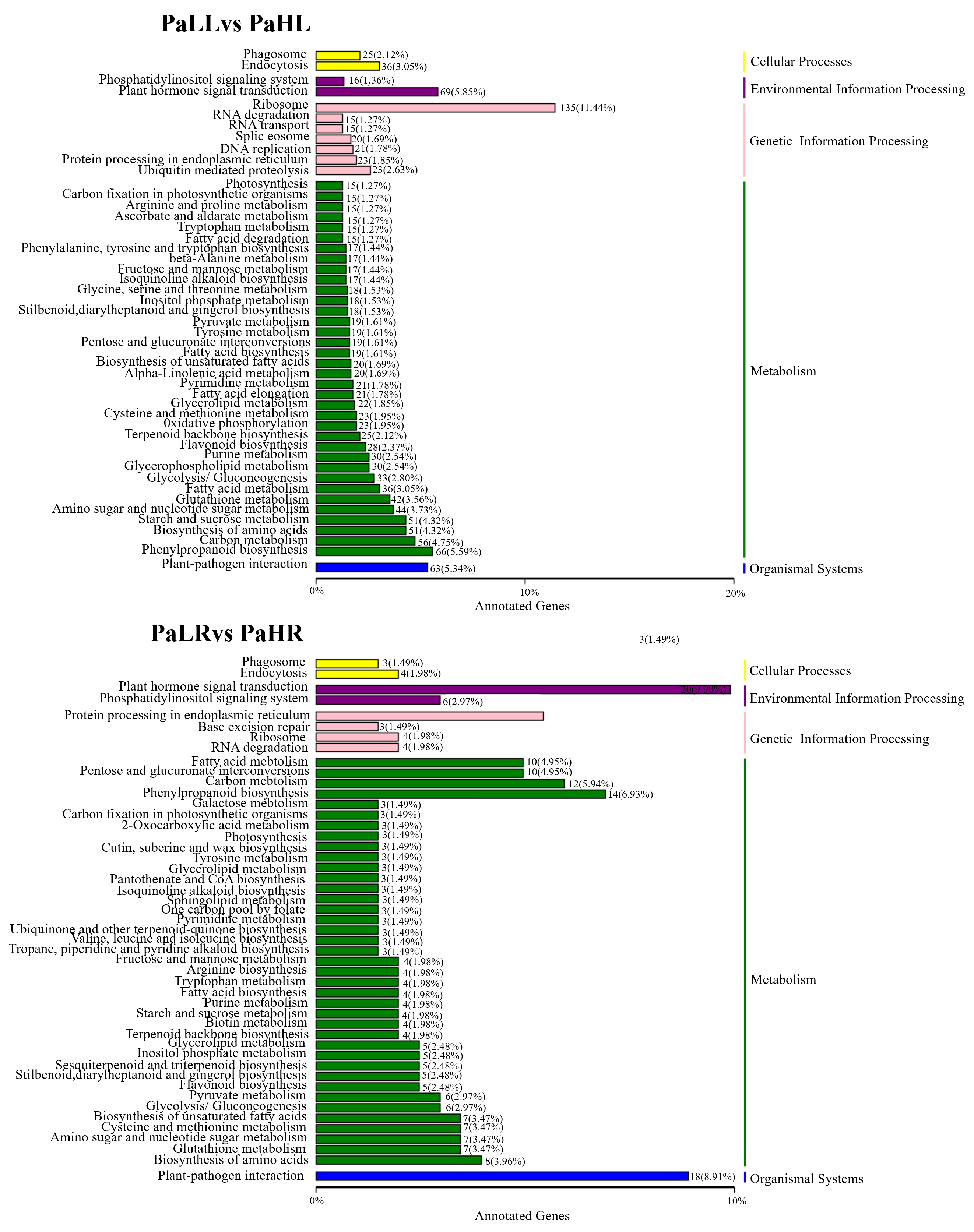

Supplement: Supplementary file 1 [file genes-15-01600-s001.zip › Figure S3.tif]
